# Structural basis of σ appropriation

**DOI:** 10.1093/nar/gkz682

**Published:** 2019-08-08

**Authors:** Jing Shi, Aijia Wen, Minxing Zhao, Linlin You, Yu Zhang, Yu Feng

**Affiliations:** 1 Department of Biophysics, and Department of Pathology of Sir Run Run Shaw Hospital, Zhejiang University School of Medicine, Hangzhou 310058, China; 2 Department of Emergency Medicine of the First Affiliated Hospital, Zhejiang University School of Medicine, Hangzhou 310003, China; 3 Key Laboratory of Synthetic Biology, CAS Center for Excellence in Molecular Plant Sciences, Shanghai Institute of Plant Physiology and Ecology, Chinese Academy of Sciences, Shanghai 200032, China; 4 University of Chinese Academy of Sciences, Beijing 100049, China

## Abstract

Bacteriophage T4 middle promoters are activated through a process called σ appropriation, which requires the concerted effort of two T4-encoded transcription factors: AsiA and MotA. Despite extensive biochemical and genetic analyses, puzzle remains, in part, because of a lack of precise structural information for σ appropriation complex. Here, we report a single-particle cryo-electron microscopy (cryo-EM) structure of an intact σ appropriation complex, comprising AsiA, MotA, *Escherichia coli* RNA polymerase (RNAP), σ^70^ and a T4 middle promoter. As expected, AsiA binds to and remodels σ region 4 to prevent its contact with host promoters. Unexpectedly, AsiA undergoes a large conformational change, takes over the job of σ region 4 and provides an anchor point for the upstream double-stranded DNA. Because σ region 4 is conserved among bacteria, other transcription factors may use the same strategy to alter the landscape of transcription immediately. Together, the structure provides a foundation for understanding σ appropriation and transcription activation.

## INTRODUCTION

Bacterial transcription is catalyzed by RNA polymerase (RNAP), which consists of α^I^, α^II^, β, β′ and ω subunits. To initiate transcription without transcription activators (referred to as canonical transcription initiation in this paper), bacterial RNAP needs to form holoenzyme with σ factor (1–10). The principle σ factor, σ^70^ in *Escherichia coli* (*E. coli*), contacts RNAP extensively and mediates sequence-specific interactions with promoter DNA. In particular, σ conserved region σR2 contacts a domain of β′ subunit known as the clamp helices and mediates sequence-specific interactions with the promoter -10 element, while σ conserved region σR4 contacts the β flap tip helix (FTH) and the β C-terminal region (βCTR), and mediates sequence-specific interaction with the promoter -35 element.

Transcription is the step at which most regulation of gene expression occurs. For many years, bacteriophage T4 has provided a simple model system to investigate mechanisms underlying this process (11,12). Because bacteriophage T4 does not encode its own RNAP, it must redirect RNAP of its host to the correct genes at the correct time. T4 middle genes encode proteins for replication, recombination and nucleotide metabolism (11). Unlike host promoters, T4 middle promoters contain a promoter -10 element and a MotA box, which is centered around position -30 and replaces the promoter -35 element (13). Transcription of T4 middle genes proceeds by activation of T4 middle promoters through a process called σ appropriation, which requires the concerted effort of two T4-encoded transcription factors: AsiA and MotA (11,14). In this process, the co-activator AisA binds to and remodels σR4, which then allows the activator MotA to interact with σR4, as well. In addition, the conformational change of σR4 prevents its normal contact with the promoter -35 element, so the transcription of host genes is inhibited.

The structural basis of σ appropriation has been studied extensively. Available structures include AsiA alone and in complex with σR4 (15–17), C-terminal domain of MotA (MotA^CTD^) alone and in complex with DNA (18,19), and N-terminal domain of MotA (MotA^NTD^) (20,21). Even a holistic model for σ appropriation complex was developed using experimentally determined structures, biochemically observed restrains and molecular modeling (22). Nevertheless, puzzle remains, in part, because of a lack of precise structural information for σ appropriation complex. For example, although the significance of the N-terminal region of AsiA in σ appropriation is conclusive (15,23,24), the role of the C-terminal region remains controversial (22,23,25–27). Furthermore, the G1249D substitution within the β subunit specifically impairs middle promoter activation both *in vivo* and *in vitro* (22,28). However, molecular modeling suggests that the position of G1249 is far from AsiA, MotA, DNA and σR4 (22). Last but not least, AsiA contains a helix-turn-helix (HTH) motif, suggesting the possibility of an interaction between AsiA and DNA (17). However, no such interaction has been confirmed yet.

To solve these puzzles conclusively, we determined a single-particle cryo-electron microscopy (cryo-EM) structure of an intact σ appropriation complex at 3.79 Å resolution. The structure defines the protein–protein and protein–DNA interactions that mediate σ appropriation. Strikingly, AsiA alters its conformation to engage RNAP and provides an anchor point for the upstream double-stranded DNA (dsDNA).

## MATERIALS AND METHODS

### AsiA protein

Gene encoding AsiA was synthesized and subcloned to pET28a by GENEWIZ, Inc. *Escherichia coli* strain BL21(DE3) (Invitrogen, Inc.) was transformed with plasmid pET28a-NH-AsiA (GENEWIZ, Inc.) encoding N-hexahistidine-tagged AsiA under the control of the bacteriophage T7 gene 10 promoter. Single colonies of the resulting transformants were used to inoculate 1 L LB broth containing 50 μg/ml kanamycin, cultures were incubated at 37°C with shaking until OD_600_ = 0.6, cultures were induced by addition of isopropyl β-D-1-thiogalactopyranoside (IPTG) to 0.4 mM, and cultures were incubated 75 min at 30°C. Then, cells were harvested by centrifugation (5000 rpm; 10 min at 4°C), resuspended in 20 ml buffer A (20 mM Tris-HCl, pH 8.0, 0.1 M NaCl, 5% glycerol) and lysed using a JN-02C cell disrupter (JNBIO, Inc.). The lysate was centrifuged (12 000 rpm; 45 min at 4°C), and the supernatant was loaded onto a 2 ml column of Ni-NTA agarose (Qiagen, Inc.) equilibrated with buffer A. The column was washed with 10 ml buffer A containing 0.16 M imidazole and eluted with 10 ml buffer A containing 0.5 M imidazole. The sample was further purified by anion-exchange chromatography on a Mono Q 10/100 GL column (GE Healthcare, Inc.; 160 ml linear gradient of 0.1–1 M NaCl in buffer A). Fractions containing AisA were pooled and stored at −80°C. AsiA derivative was expressed and purified in the same way as wild-type protein. Yields were ∼2 mg/l, and purities were >95%.

### MotA protein

Gene encoding MotA was synthesized and subcloned to pET21a by GENEWIZ, Inc. *Escherichia coli* strain BL21(DE3) (Invitrogen, Inc.) was transformed with plasmid pET21a-MotA (GENEWIZ, Inc.) encoding MotA under the control of the bacteriophage T7 gene 10 promoter. Single colonies of the resulting transformants were used to inoculate 1 L LB broth containing 50 μg/ml ampicillin, cultures were incubated at 37°C with shaking until OD_600_ = 0.6, cultures were induced by the addition of IPTG to 1 mM, and cultures were incubated 3 h at 37°C. Then, cells were harvested by centrifugation (5000 rpm; 10 min at 4°C), resuspended in 20 ml buffer B (10 mM Tris-HCl, pH 7.5, 0.2 M NaCl, 5% glycerol, 1 mM EDTA and 1 mM dithiothreitol (DTT)) and lysed using a JN-02C cell disrupter (JNBIO, Inc.). The lysate was centrifuged (12 000 rpm; 45 min at 4°C), and the supernatant was loaded onto a 5 ml column of HiTrap Heparin HP (GE Healthcare, Inc.) equilibrated in buffer B and eluted with a 100 ml linear gradient of 0.2–1 M NaCl in buffer B. The sample was further purified by cation-exchange chromatography on a Mono S 10/100 GL column (GE Healthcare, Inc.; 160 ml linear gradient of 0.2–1 M NaCl in buffer B). Fractions containing MotA were pooled and stored at −80°C. Yields were ∼2 mg/l, and purities were >95%.

### *Escherichia coli* σ^70^

*Escherichia coli* σ^70^ was prepared using plasmid pGEMD (29) as reported (7). Yield was ∼50 mg/l, and purity was >95%.

### *Escherichia coli* RNAP core enzyme

*Escherichia coli* RNAP core enzyme was prepared from *E. coli* strain BL21(DE3) (Invitrogen, Inc.) transformed with plasmid pIA900 (30), using culture, induction and purification procedures essentially as reported (7). Yield was ∼2.5 mg/l, and purity was >95%.

### Assembly of σ appropriation complex

**Figure 1.**
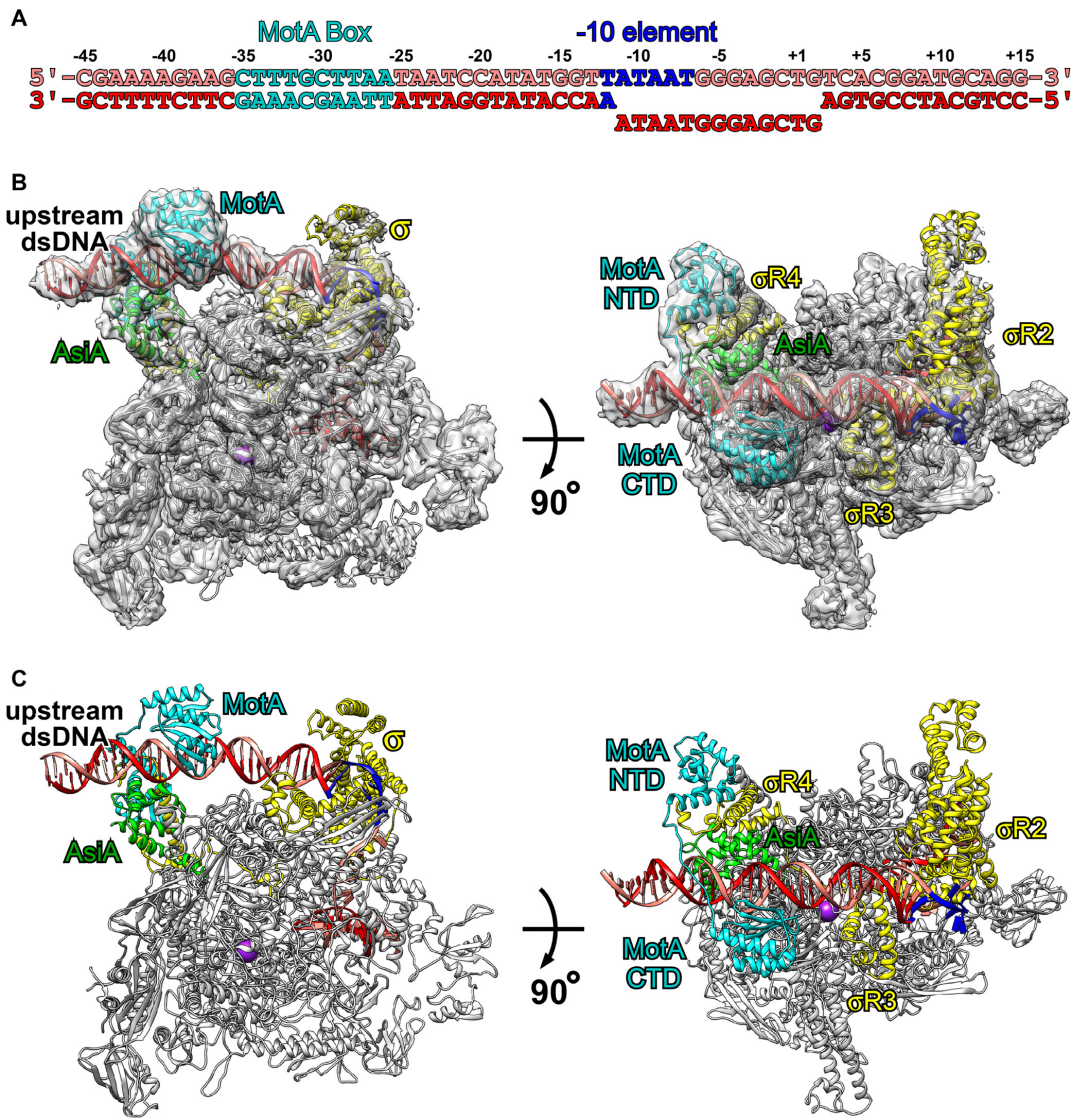
Cryo-EM structure of σ appropriation complex. (**A**) Nucleic-acid scaffold sequence used for cryo-EM. Salmon, non-template strand DNA; red, template strand DNA; cyan, MotA box; blue, the promoter -10 element. Positions are numbered relative to the transcription start site. (**B**) The cryo-EM density map without B-factor sharpening and the superimposed model of σ appropriation complex. Gray, RNAP core enzyme; yellow, σ^70^; cyan, MotA; green, AsiA; violet, active center Mg^2+^; salmon, non-template strand DNA; red, template strand DNA; blue, the promoter -10 element. (**C**) The model of σ appropriation complex. View orientations and colors as in (B).

DNA oligonucleotides (sequences in Figure 1A) (Sangon Biotech, Inc.) were dissolved in nuclease-free water to ∼1 mM and stored at −80°C. Template strand DNA and non-template strand DNA were annealed at a 1:1 ratio in 10 mM Tris-HCl, pH 7.9, 0.2 M NaCl and stored at −80°C. σ appropriation complex was prepared in reaction mixtures containing (500 μl): 9 μM σ^70^, 18 μM AsiA, 4.5 μM *E. coli* RNAP core enzyme, 5 μM DNA scaffold and 18 μM MotA. σ^70^ was incubated with AsiA for 10 min at 37°C, incubated with core for 10 min at 37°C and incubated with MotA and DNA scaffold for 10 min at 37°C. The mixture was applied to a Superose 6 Increase 10/300 GL column (GE Healthcare, Inc.) equilibrated in 10 mM HEPES, pH 7.5, 50 mM KCl, and the column was eluted with 24 ml of the same buffer. Fractions were checked by SDS-PAGE and the peak containing σ appropriation complex was concentrated to 20 μM using an Amicon Ultra-0.5mL centrifugal filter (10 kDa MWCO; Merck Millipore, Inc.).

### *In vitro* transcription assay of σ appropriation complex

*In vitro* transcription assay was performed in reaction mixtures containing (20 μl): 0 or 50 nM σ appropriation complex, 5 μM ATP, 5 μM CTP, 33 nM [α-^32^P]UTP (100 Bq/fmol), 40 mM Tris-HCl, pH 8.0, 75 mM NaCl, 5 mM MgCl_2_, 12.5% glycerol, 2.5 mM DTT and 50 μg/ml BSA. Reaction mixtures were incubated 10 min at 37°C. Reactions were terminated by adding 10 μl loading buffer and boiling for 5 min. Products were applied to 15% urea-polyacrylamide slab gel (19:1 acrylacmide/bisacrylamide), electrophoresed in 90 mM Tris-borate, pH 8.0 and 0.2 mM EDTA, and analyzed by storage-phosphor scanning (Typhoon; GE healthcare, Inc.)

### Cryo-EM grid preparation

Immediately before freezing, 8 mM CHAPSO was added to the sample. C-flat grids (CF-1.2/1.3-4C; Protochips, Inc.) were glow-discharged for 60 s at 15 mA prior to the application of 3 μl of the complex, then plunge-frozen in liquid ethane using a Vitrobot (FEI, Inc.) with 95% chamber humidity at 10°C.

### Cryo-EM data acquisition and processing

The grids were imaged using a 300 kV Titan Krios (FEI, Inc.) equipped with a K2 Summit direct electron detector (Gatan, Inc.). Images were recorded with Serial EM (31) in counting mode with a physical pixel size of 1.307 Å and a defocus range of 1.5–2.5 μm. Data were collected with a dose of 8 e/pixel/s. Images were recorded with a 12 s exposure and 0.25 s subframes to give a total dose of 56 e/Å^2^. Subframes were aligned and summed using MotionCor2 (32). The contrast transfer function was estimated for each summed image using CTFFIND4 (33). From the summed images, ∼10 000 particles were manually picked and subjected to 2D classification in RELION (34). 2D averages of the best classes were used as templates for auto-picking in RELION. Auto-picked particles were manually inspected, then subjected to 2D classification in RELION. Poorly populated classes were removed, resulting in a dataset of 654 794 particles. These particles were 3D classified in RELION using a map of *E. coli* elongation complex (EMD-8585) (35) low-pass filtered to 40 Å resolution as a reference. 3D classification resulted in 4 classes. Particles in Class 3 and Class 4 were 3D auto-refined, then subjected to 3D classification focused on the MotA box. From this classification, the best-resolved class containing 105 108 particles was 3D auto-refined and post-processed in RELION.

### Cryo-EM model building and refinement

The models of RNAP core enzyme, σR2 and σR3 from the structure of *E. coli* RPo (PDB 6CA0) (9), the NMR structure of AsiA-σR4, the crystal structure of MotA^NTD^ and the crystal structure of MotA^CTD^-MotA box were fitted into the cryo-EM density map using Chimera (36). The model of nucleic acids was built manually in Coot (37). The coordinates were real-space refined with secondary structure restraints in Phenix (38).

### Electrophoretic mobility shift assay to quantify σ appropriation complex

Template strand DNA oligonucleotide (5′-CACGTTTATGTGATGGTTTATTTCTATTATAACCATATGGATTATTAAGCAAAGCTTCTTTTCG-3′, Sangon Biotech, Inc.) and non-template strand DNA oligonucleotide (5′-CGAAAAGAAGCTTTGCTTAATAATCCATATGGTTATAATAGAAATAAACCATCACATAAACGTG-3′, Sangon Biotech, Inc.) were annealed at a 1:1 ratio in 10 mM Tris-HCl, pH 7.9, 0.2 M NaCl and stored at −80°C. Electrophoretic mobility shift assays were performed in reaction mixtures containing (20 μl): 0.2 μM σ^70^, 0.4 μM AsiA or AsiA derivative, 0.1 μM *E. coli* RNAP core enzyme, 0.05 μM DNA scaffold, 0.1 μM MotA, 0.1 mg/ml heparin, 7 mM Tris-HCl (pH 7.9), 50 mM Tris-Ac (pH 7.9), 0.19 M KGlu, 5 mM MgAc_2_, 0.4 mM EDTA, 0.2 mM DTT, 0.125 mg/ml BSA, 50 mM potassium phosphate (pH 6.5), 0.14 M NaCl and 22% glycerol. σ^70^ was incubated with AsiA or AsiA derivative for 10 min at 37°C, incubated with core for 10 min at 37°C, incubated with MotA and DNA scaffold for 10 min at 37°C, and incubated with 0.1 mg/ml heparin for 1 min at 37°C. The reaction mixtures were applied to 5% polyacrylamide slab gels (29:1 acrylamide/bisacrylamide), electrophoresed in 90 mM Tris-borate, pH 8.0, and 0.2 mM EDTA, stained with 4S Red Plus Nucleic Acid Stain (Sangon Biotech, Inc.) according to the procedure of the manufacturer, and analyzed by ImageJ (https://imagej.nih.gov/ij/).

### Fluorescence polarization assays of AsiA-DNA interaction

5′ 6-FAM labeled DNA oligonucleotide and unmodified DNA oligonucleotide were purchased from Sangon Biotech, Inc. Cytosine hydroxymethylated DNA oligonucleotide was purchased from GenScript, Inc. Template strand DNA oligonucleotide and non-template strand DNA oligonucleotide (sequences in Figure 4B) were annealed at a 1:1 ratio in 10 mM HEPES, pH 7.5, 50 mM KCl. 5-Hydroxymethylated cytosines within the DNA scaffold were glucosylated by treatment with β-glucosyltransferase (NEB, Inc.) according to the company protocol and purified using standard phenol–chloroform extraction followed by ethanol precipitation. Mass spectrometry confirmed that all cytosines within the DNA scaffold are hydroxymethylated and glucosylated. Equilibrium fluorescence polarization assays were performed in a 96-well microplate format. Reaction mixtures contained (100 μl): 0–100 μM AsiA or AsiA derivative, 0.1 μM 6-FAM-labelled DNA scaffold, 10 mM HEPES, pH 7.5, 50 mM KCl. Following incubation mixtures for 10 min at 25°C, fluorescence emission intensities were measured using a SpectraMax M5 microplate reader (Molecular Devices, Inc.; excitation wavelength = 494 nm; emission wavelength = 518 nm). Fluorescence polarization was calculated using:

(1) $$\begin{equation*}{{P }} = {\rm{ }}( {{I_{{\rm VV}}} - {I_{{\rm VH}}}})/( {{I_{{\rm VV}}} + {I_{{\rm VH}}}})\end{equation*}$$

where *I*_VV_ and *I*_VH_ are fluorescence intensities with the excitation polarizer at the vertical position and the emission polarizer at, respectively, the vertical position and the horizontal position.

Equilibrium dissociation constant, *K*_D_, was extracted by non-linear regression using the equation:

(2) $$\begin{equation*}{{P = }}{{{P}}_{\rm{f}}}{\rm{ + }}\{ {( {{{{P}}_{\rm{b}}}{\rm{ - }}{{{P}}_{\rm{f}}}})\times [ {{A}}]{\rm{ / }}( {{{{K}}_{\rm{D}}}{\rm{ + }}[ {{A}}]} )}\}\end{equation*}$$

where *P* is the fluorescence polarization at a given concentration of AsiA, *P*_f_ is the fluorescence polarization for free 6-FAM-labelled DNA scaffold, *P*_b_ is the fluorescence polarization for bound 6-FAM labeled DNA scaffold and [*A*] is the concentration of AsiA or AsiA derivative.

## RESULTS

### Overall structure of σ appropriation complex

To obtain a structure of σ appropriation complex, we used a nucleic-acid scaffold corresponding to positions −45 to +15 of a T4 middle promoter (*P*_uvsX_, positions numbered relative to the transcription start site; Figure 1A). The scaffold contains a MotA box centered between positions −30 and −31, a consensus promoter -10 element and a 13-bp transcription bubble maintained in the unwound state by having non-complementary sequences on non-template and template strands.

First, σ^70^ was incubated with excessive AsiA to ensure that there was no free σ^70^. Then, RNAP core enzyme was added to form AsiA-associated RNAP. Finally, MotA and the nucleic-acid scaffold were added to form σ appropriation complex, which was purified by gel filtration (Supplementary Figure S1A). SDS-PAGE confirmed that all protein components are present in the complex with stoichiometric levels (Supplementary Figure S1B). Nucleic-acid staining confirmed that the complex contains DNA scaffold (Supplementary Figure S1C). *In vitro* transcription assay with ATP, CTP and UTP confirmed that the complex is capable of transcription initiation (Supplementary Figure S1D).

We then froze the complex, collected data on Titan Krios and determined the structure at a nominal resolution of 3.79 Å (Figure 1B and C; Supplementary Figures S2 and S3; Supplementary Table S1). Local resolution calculation indicates that the central core of the structure was determined to 3.4–4.0 Å resolution (Supplementary Figure S3C).

The RNAP, σR2 and σR3 of the structure are very similar to the previously reported *E. coli* RNAP–promoter open complex (RPo) structure (9) with root-mean-square deviation (RMSD) of 1.284 Å (3551 Cαs aligned). Moreover, σR2 and σR3 interact with the promoter -10 element and extended -10 element in the same way as in RPo, in accordance with the observation that the protein–DNA interactions downstream of position -20 are not affected by the presence of AsiA and MotA (14). It is worth noting that nearly all T4 middle promoters have a stringent requirement for an excellent match to the consensus promoter -10 element and about half of the middle promoters also contain a consensus extended -10 element (11).

### Both σR3.2 and σR4 are remodeled by AsiA

**Figure 2.**
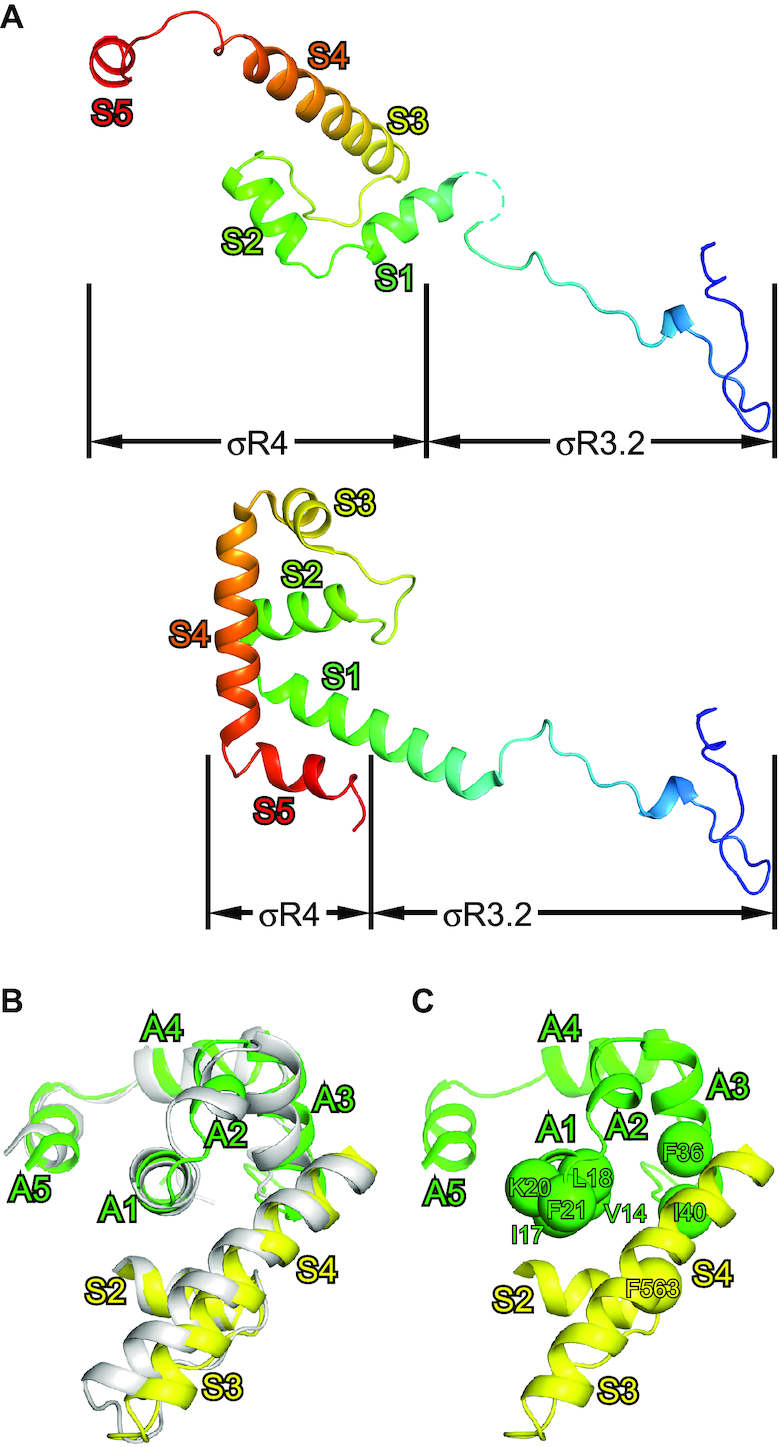
Both σR3.2 and σR4 are remodeled by AsiA. (**A**) Comparison of σR3.2 and σR4 in σ appropriation complex (upper subpanel) and in RPo (PDB 6CA0; lower subpanel). (**B**) Superimposition of AsiA–σR4 in σ appropriation complex on the NMR structure of AsiA–σR4 (PDB 1TLH). (**C**) Residues on AsiA–σR4 interface have been confirmed to impair AsiA’s function. View orientation as in (B). The α-carbons of these residues are shown as spheres.

Compared with the structure of RPo, the conformation of the C-terminal helix of σR3.2 and σR4 in σ appropriation complex is dramatically different (Figure 2A and Supplementary Figure S4A). The C-terminal helix of σR3.2 (530-543) and the first helix of σR4 (S1) form a continuous helix in RPo. However, most residues of the C-terminal helix of σR3.2 (532-539) become disordered in σ appropriation complex probably due to clash with the C-terminal helix of AsiA (A6; see below). σR4 adopts an elongated conformation with all helices completely reorganized. For example, the third and forth helices (S3 and S4) of σR4 and the loop connecting them, which constitute the HTH motif and participate in binding the -35 element in RPo, are converted into one continuous helix in σ appropriation complex.

Early work indicated that AsiA binds very tightly to σR4 and a NMR structure of AsiA in complex with σR4 has been determined (15). The structure of AsiA and σR4 in σ appropriation complex is superimposable on the NMR structure (neglecting A6 of AsiA and S5 of σR4, which do not contribute to AsiA–σR4 interaction; Figure 2B), indicating that AsiA makes a similar set of interactions in both structures. Substitutions of some of the interacting residues (Figure 2C) have been demonstrated to impair AsiA’s function (23,24,39–41), verifying that the cryo-EM structure is biologically relevant. The AsiA–σR4 interface area is large (1194 Å^2^), which explains the tight binding between AsiA and σ and emphasizes the critical role of AsiA in the remodeling of σR4.

### AsiA alters its conformation to engage RNAP

**Figure 3.**
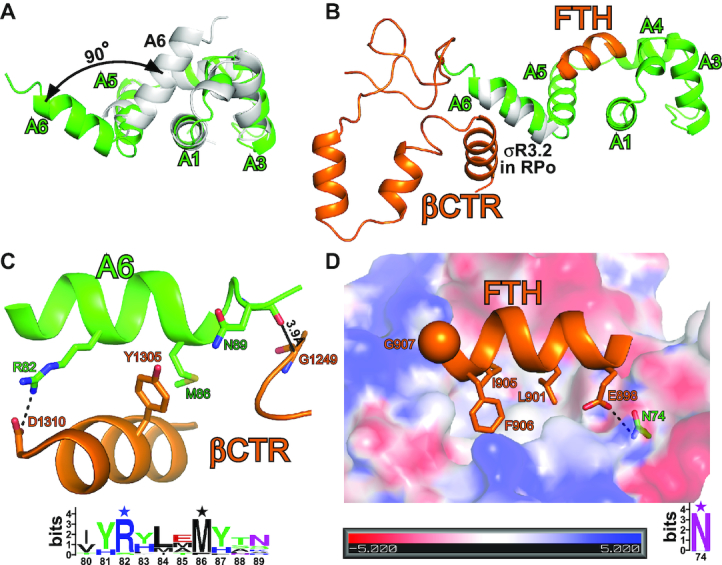
AsiA alters its conformation to engage RNAP. (**A**) A6 is rotated by ∼90°, about a ‘pivot’ formed by the short loop between A5 and A6. (**B**) A6 replaces the C-terminal helix of σR3.2 and contacts βCTR, while FTH binds to AsiA. (**C**) The interactions between A6 and βCTR. The salt bridge (≤4.5 Å) is denoted as a dashed line. 39 sequences were found and aligned on UniProt (www.uniprot.org). All the sequences are derived from *Tevenvirinae* and annotated as anti-σ protein. The sequence logo was generated on the WebLogo server (54) and contact residues are highlighted by stars above the sequence. (**D**) FTH binds to a hydrophobic pocket on AsiA. The α-carbon of β residue G907 is shown as a sphere. The sequence logo was generated as in (C).

Strikingly, whereas in the NMR structure of AsiA in complex with σR4, A6 binds to a pocket on the remainder of AsiA, in σ appropriation complex, A6 is rotated by ∼90°, about a ‘pivot’ formed by the short loop between A5 and A6, inserted into the RNA exit channel in the same orientation as the C-terminal helix of σR3.2 in RPo, and contacted by βCTR (Figure 3A and B; Supplementary Figure S4B). Specifically, AsiA-conserved residue R82 forms a salt bridge with β residue D1310 (Figure 3C and Supplementary Figure S4C), consistent with the previous report that MotA-dependent transcription activation was severely compromised by charge-reversal substitution of AsiA residue R82 (26). In addition, AsiA conserved residue M86 makes van der Waals interactions with β residue Y1305, consistent with the observation that AsiA substitution M86T also compromises MotA-dependent transcription activation (26).

The G1249D substitution within the β subunit specifically impairs middle promoter activation both *in vivo* and *in vitro* (22,28). In our structure, the distance between the α-carbon of β residue G1249 and the carbonyl oxygen of AsiA residue N89 is 3.9 Å (Figures 3C and Supplementary figure S4C). We infer that the G1249D substitution would introduce steric clash between the side chain of the aspartic acid residue and the carbonyl oxygen of N89 and be incompatible with the current conformation of σ appropriation complex. In accordance with our inference, benzoyl-phenylalanine (BpA) photo-crosslinking experiments showed that even though RNAP bearing G1249D substitution within the β subunit formed an AsiA-associated complex, the conformation of the complex differed from that formed by wild-type RNAP (22).

In σ appropriation complex, FTH binds the hydrophobic pocket of AsiA, which is occupied by A6 in the NMR structure (Figure 3D and Supplementary figure S4D). In particular, three hydrophobic residues of FTH (L901, I905 and F906) insert deeply into the pocket, which is consistent with the previous report that amino acid substitutions I905A and F906A in combination strongly disrupt the AsiA–β flap interaction (27). Additionally, the N74D substitution of AsiA and the G907K substitution of FTH that were reported to jeopardize AsiA–β flap interaction (27) can be interpreted by our structure. In our structure, AsiA conserved residue N74 forms an H-bond with FTH residue E898, and FTH residue G907 is close to a patch of positively charged residues on AsiA. The N74D substitution of AsiA would disrupt the H-bond and cause electrostatic repulsion between the aspartic acid residue and FTH residue E898. Similarly, the G907K substitution of FTH would cause electrostatic repulsion between the lysine residue and the patch of positively charged residues on AsiA.

### The HTH motif of AsiA contacts the upstream dsDNA

**Figure 4.**
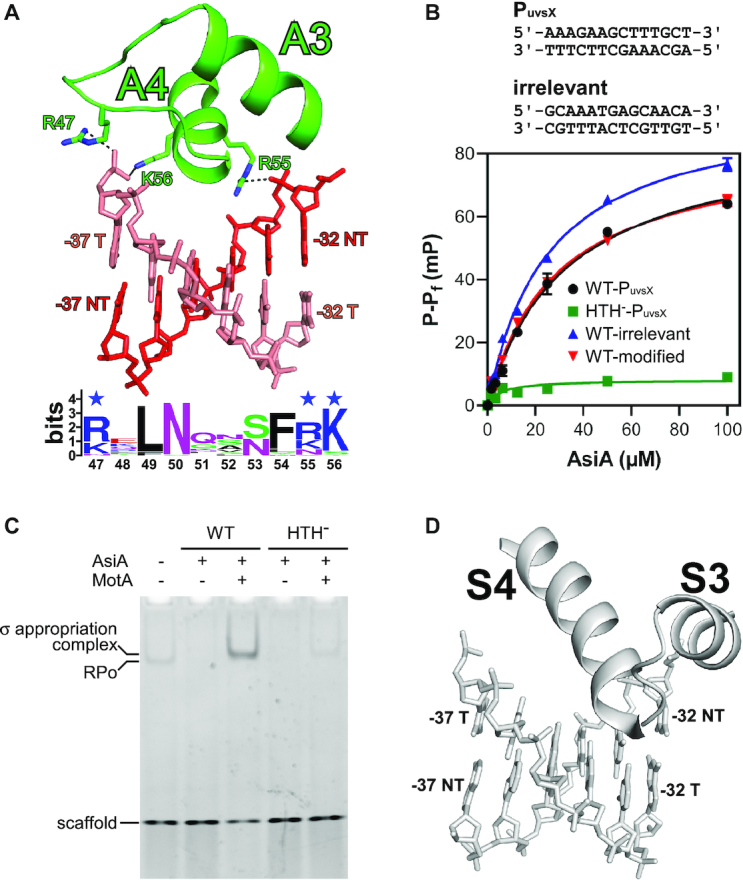
The HTH motif of AsiA contacts the upstream dsDNA. (**A**) The interaction between AsiA and the upstream dsDNA. Salt bridges (≤4.5 Å) are denoted as dashed lines. The sequence logo was generated as in Figure 3C. (**B**) Effects on AsiA–DNA binding affinity of substituting HTH residues (HTH^−^) or using different DNA scaffolds (mean ± SEM; three determinations). The sequences of the DNA scaffolds are shown above the chart. (**C**) Effects on the formation of RPo and σ appropriation complex of substituting HTH residues (HTH^−^). (**D**) In RPo (PDB 6CA0), S4 of σR4 inserts into the major groove of the promoter -35 element to read out sequence.

The NMR structure of AsiA shows that A3, A4 and the loop connecting them constitute a HTH motif (15–17). In our structure, the HTH motif contacts the upstream portion of the MotA box with A4 inserting into the major groove of DNA between positions -32 and -37 (Figure 4A and Supplementary figure S4E). In particular, two conserved positively charged residues (R55 and K56) on A4 and one conserved positively charged residue (R47) on the loop form three salt bridges with the DNA backbone phosphates. In accordance, alanine substitution of these residues in combination (HTH^−^) eliminates AsiA–DNA interaction (Figures 4B).

Previous studies have shown that *P*_uvsX_ can be transcribed by RNAP alone, which is inhibited by AsiA in the absence of MotA and activated by the presence of both AsiA and MotA (23). In accordance with the previous studies, gel shift assay shows that RPo is formed by RNAP alone (Figure 4C, lane 1). AsiA inhibits the formation of RPo (Figure 4C, lane 2), while AsiA and MotA in combination promotes the formation of σ appropriation complex, which migrates slower than RPo on native gel (Figure 4C, lane 3). AsiA (HTH^−^) inhibits the formation of RPo as efficiently as the wild-type protein (Figure 4C, lane 4), indicating that AsiA (HTH^−^) is well folded and the AsiA–DNA interaction is not required for its transcription inhibition activity. AsiA (HTH^−^) forms less σ appropriation complex than the wild-type protein (Figure 4C, lane 5), indicating that the AsiA–DNA interaction is important for its transcription activation activity.

For canonical transcription initiation, S4 of σR4 inserts into the major groove of the promoter -35 element to read out sequence (Figure 4D). Compared with S4, A4 does not insert so deeply and can not read out the bases in the major groove, which is consistent with the observation that the sequences between positions -32 and -37 are not conserved in T4 middle promoters (42–45). To further confirm that the AsiA–DNA interaction is non-sequence-specific, we tested the interaction between AsiA and an irrelevant DNA scaffold using fluorescence polarization assay. It turned out that AsiA binds the irrelevant DNA scaffold with comparable affinity (Figure 4B).

Each cytosine in T4 DNA is hydroxymethylated and glucosylated, which places a bulky group within the major groove of T4 DNA (11,19). We infer that A4 of AsiA inserts shallowly into the major groove of T4 DNA, so it will not clash with the glucosylated hydroxymethyl moiety. In order to test this hypothesis experimentally, we prepared hydroxymethylated and glucosylated DNA scaffold using β-glucosyltransferase and confirmed its binding with AsiA using fluorescence polarization assay (Figure 4B).

### MotA^NTD^ contacts both S4 and S5 of σR4

**Figure 5.**
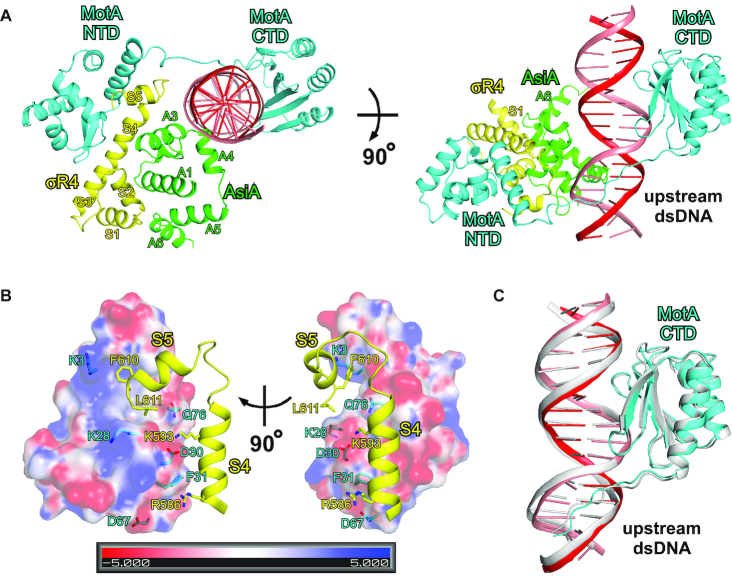
MotA^NTD^ contacts both S4 and S5 of σR4. (**A**) σR4 is sandwiched between MotA^NTD^ and AsiA, while the upstream dsDNA is sandwiched between MotA^CTD^ and AsiA. (**B**) Interactions between MotA^NTD^ and σR4. (**C**) Superimposition of MotA^CTD^, the linker and the MotA box in σ appropriation complex on the crystal structure of MotA–MotA box (PDB 5JLT).

MotA contains two domains connected by a flexible linker. In the structure of σ appropriation complex, the trans-activation domain, MotA^NTD^, interacts with the remodeled σR4, while, the DNA binding domain, MotA^CTD^ binds the MotA box (Figure 5A and Supplementary Figure S4F). In other words, σR4 is sandwiched between MotA^NTD^ and AsiA, while the upstream dsDNA is sandwiched between MotA^CTD^ and AsiA.

The structure of MotA^NTD^ in σ appropriation complex is very similar to the previously reported crystal structure and NMR structure of MotA^NTD^ (20,21), with root-mean-square deviation (RMSD) of 0.879 Å (89 Cαs aligned) and 1.719 Å (89 Cαs aligned), respectively. S5 of σR4 binds to a basic/hydrophobic groove on MotA^NTD^ (Figure 5B), which is consistent with the previous report that mutation of S5 residues F610 and L611 impairs both MotA binding to σ^70^ and MotA-dependent transcription activation (46). The structure is also consistent with the observation that mutation of MotA residues K3, K28 and Q76, which lie in the basic/hydrophobic groove, impairs the ability of MotA to interact with S5 and to activate transcription (47). Besides the interaction of S5, basic residues on S4 interact with an acidic/hydrophobic surface on MotA^NTD^ and mutation of MotA residues D30, F31 and D67, which lie on the acidic/hydrophobic surface, also have deleterious effects on the interaction with σ^70^, MotA-dependent transcription activation and phage viability (20,47).

Because of the glucosylated hydroxymethyl moiety in the major groove, a HTH motif cannot be used to read out sequence from the major groove. Therefore, a novel strategy is utilized by T4. MotA^CTD^ adopts a saddle-shaped, ‘double wing’ conformation, straddles the major groove and leaves enough space to accommodate a glucosylated hydroxymethyl moiety, while the linker engages the minor groove in the upstream portion of the MotA box (Figure 5A). Structural modeling based on biochemical data has predicted that MotA linker engages the minor groove in the upstream portion of the MotA box (22), which was further confirmed by the crystal structure of MotA in complex with MotA box (19). The structure of MotA^CTD^, the linker and the MotA box in σ appropriation complex is superimposable on the crystal structure of MotA in complex with MotA box (Figure 5C), indicating that MotA makes a similar set of interactions in both structures.

## DISCUSSION

Previous work has shown that AsiA containing either a deletion of the C-terminal half of AsiA (23) or a deletion of A6 (22,23) is still active *in vivo* and *in vitro*. Other work has shown that the AsiA ortholog within the bacteriophage KVP40 can substitute for T4 AsiA in transcription, even though there is practically no homology between these AsiA proteins after residue 60 (25). According to our structure, AsiA engages RNAP through two sets of interactions. First, σ^70^, which is anchored to RNAP through σR2 and σR3, grabs the N-terminal half of AsiA through σR4. Second, the C-terminal half of AsiA interacts with the βCTR and FTH of RNAP directly. If the first set of interactions are strong enough to hold AsiA and RNAP together, disruption of the second set of interactions will not necessarily eliminate the formation of σ appropriation complex. To test this possibility, we truncated the C-terminus of AsiA (ΔA6) and tested σ appropriation complex formation with the mutant protein (Supplementary Figure S5). As expected, truncation of the C-terminus of AsiA affects the formation of σ appropriation complex, but there is still a notable level of σ appropriation complex. Therefore, we infer that the notable level of σ appropriation complex is enough for the transcription of T4 middle genes *in vivo* and *in vitro*.

Despite the absence of sequence homology or structural similarity except for the HTH motif, the function of AsiA is analogous to that of σR4 during canonical transcription initiation. During canonical transcription initiation, σR4 contacts the FTH and the βCTR, and mediates sequence-specific interaction with the promoter -35 element (Supplementary Figure S6A). As a mimic of σR4, AsiA also contacts the FTH and the βCTR, and provides an anchor point for the upstream dsDNA (Supplementary Figure S6B). The anchoring of the upstream dsDNA by AsiA may compensate for the weak binding between MotA and MotA box (11).

Extensive biochemical studies with T4 middle promoter P_rIIB2_ have been conducted in order to unveil the mechanism of MotA-dependent transcription activation (48,49). Consistent with the cryo-EM structure, surface plasmon resonance experiments proved that MotA promotes the formation of RNAP-promoter closed complex (RPc), but not the transition from RPc to RPo (48). Further consistent with the cryo-EM structure, *in vitro* transcription experiments indicated that MotA and AsiA work together to facilitate promoter escape (48), which can be attributed to the conformational change of the C-terminal helix of σR3.2 and σR4.

Compared with classical transcription activation, σ appropriation is more efficient. Typical transcription activators, such as class I and class II transcription activators, function by improving target promoters, but they have little effect on other promoters (7,50–53). On the contrary, AsiA and MotA shut down the majority of cellular genes, whose transcription requires σ^70^, and devote most resources to one set of promoters. This system potentially could be engineered to change the landscape of transcription with high efficiency in synthetic biology.

In conclusion, the structure of σ appropriation complex defines the mechanisms by which σR4 can be remodeled and then exploited to alter promoter specificity. Because σR4 is a conserved feature of all bacterial RNAPs (1–10), other examples of σ appropriation may emerge in the future.

## DATA AVAILABILITY

The accession number for the cryo-EM density map reported in this paper is Electron Microscopy Data Bank: EMD-9916. The accession numbers for the atomic coordinates reported in this paper are Protein Date Bank: 6K4Y.

## Supplementary Material

gkz682_Supplemental_File
