# Supplementary material for: Structural basis of σ appropriation
Source: Nucleic Acids Res. 2019 Aug 8;47(17):9423–32. doi: 10.1093/nar/gkz682 (PMC6755090; doi:10.1093/nar/gkz682)
Supplement: gkz682_Supplemental_File [file gkz682_supplemental_file.pdf]

# Structural basis of σ appropriation

Shi et al.

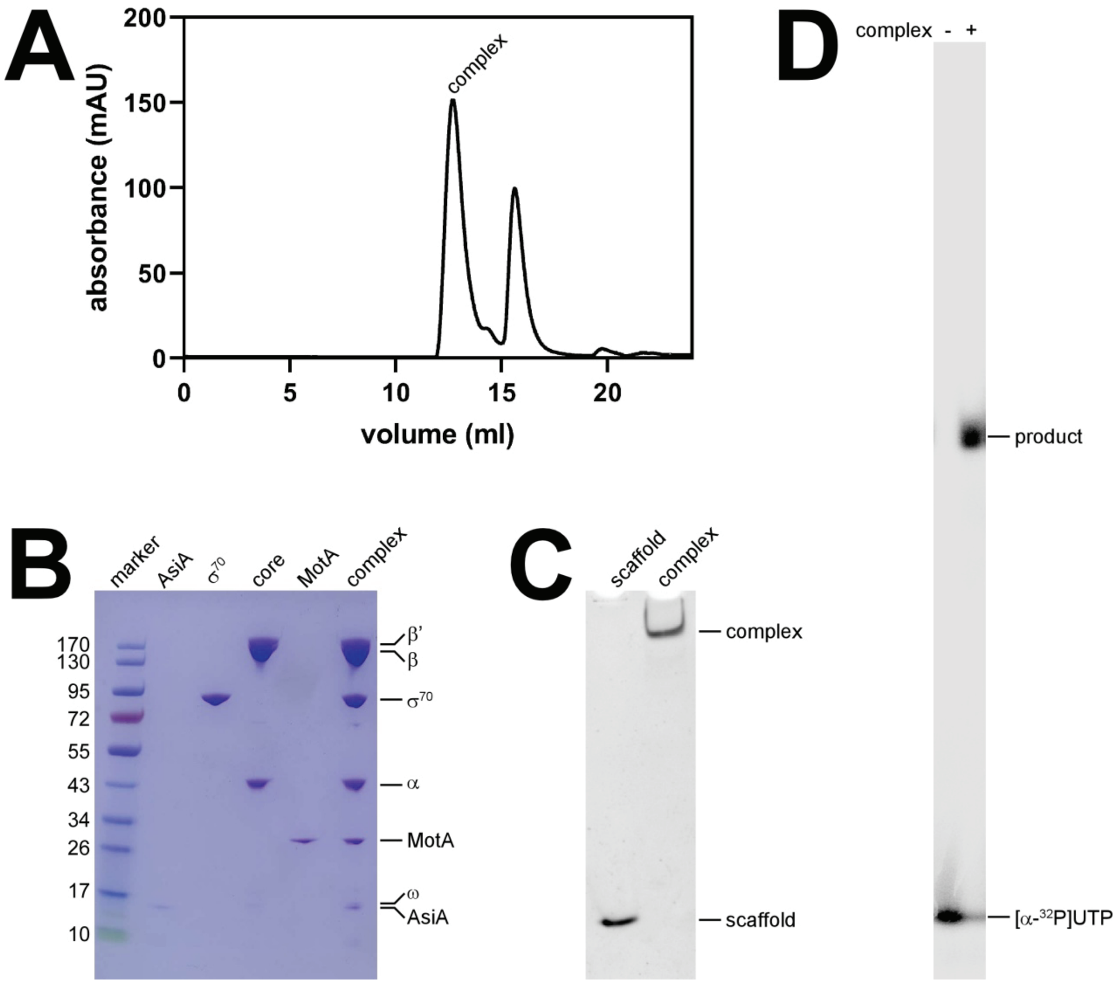


**Figure S1. Purification and verification of σ appropriation complex.**

(A) Chromatogram of gel filtration.

(B) SDS-PAGE of the purified complex. 16 pmoles of protein or complex were loaded each lane.

(C) Native gel of the purified complex. The gel was stained with 4S Red Plus Nucleic Acid Stain (Sangon Biotech, Inc.) according to the procedure of the manufacturer.

(D) *In vitro* transcription assay with ATP, CTP, and UTP confirmed that the complex is capable of transcription initiation.

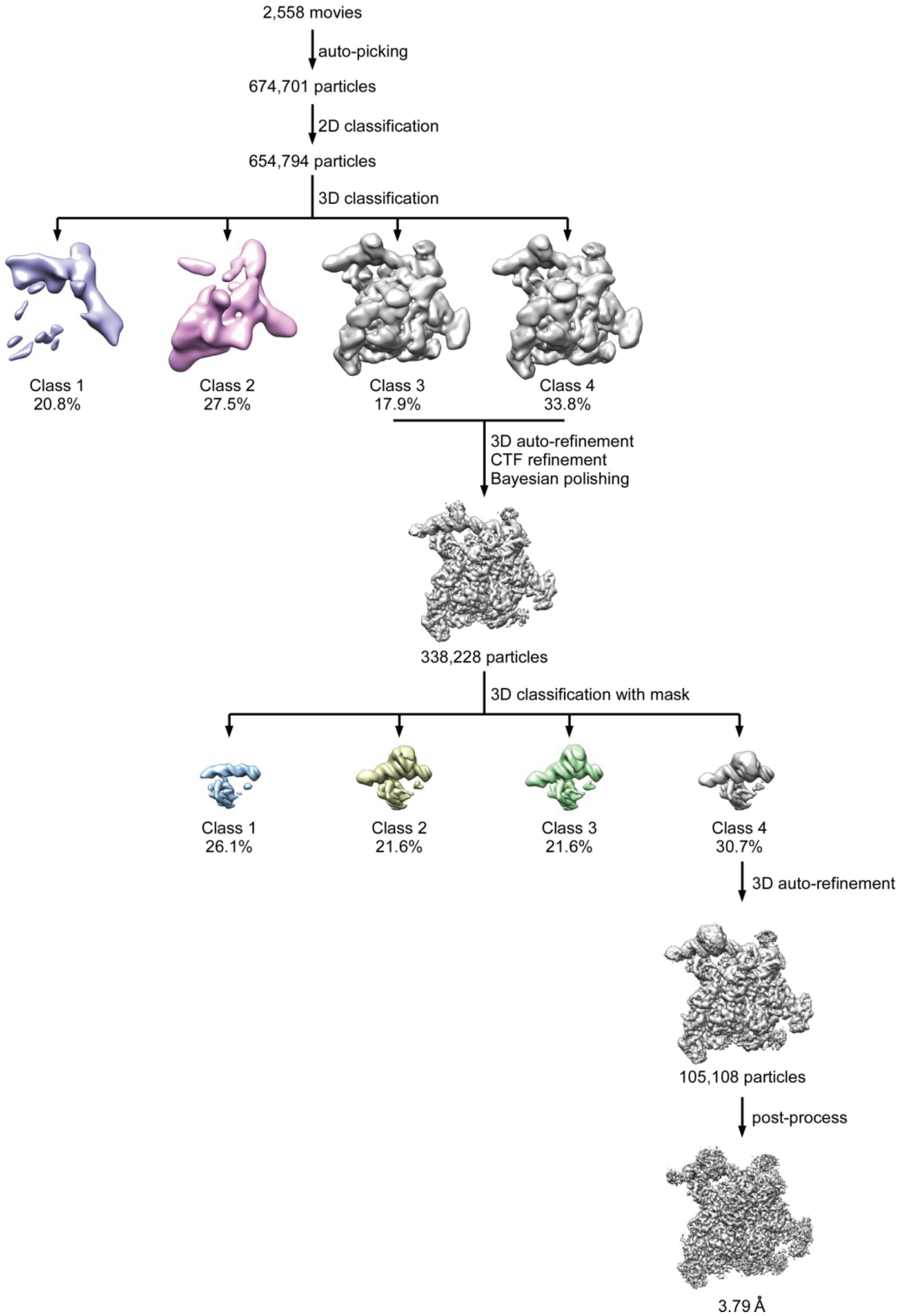


**Figure S2. Data processing pipeline for the cryo-EM data.**

3D classification focused on the MotA box resulted in 4 classes. Class 1 does not contain MotA, while the other classes contain MotA. Nevertheless, AsiA adopts the same conformation in all classes. Because the upstream dsDNA are bent to different extent, the AsiA-DNA interactions are not exactly the same in different classes, which is consistent with the hypothesis that AsiA-DNA interaction is non-sequence-specific. We chose Class 4 for further refinement because it represents the most abundant population.

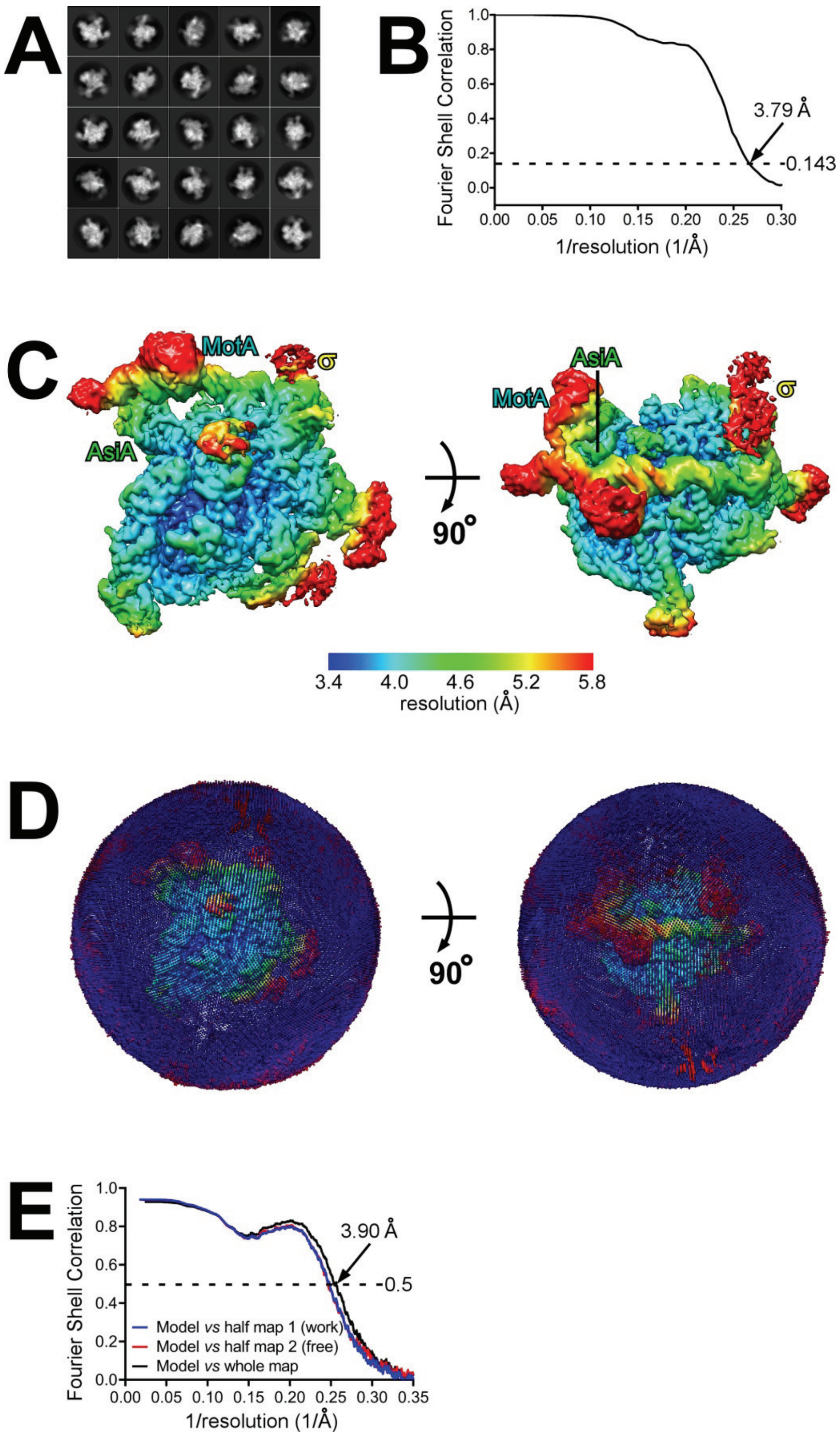


**Figure S3. Cryo-EM of σ appropriation complex.**

(A) Representative classes from 2D classification.

(B) Gold-standard FSC. The gold-standard FSC was calculated by comparing the two independently determined half-maps from RELION. The dashed line represents the 0.143 FSC cutoff, which indicates a nominal resolution of 3.79 Å.

(C) Cryo-EM density map colored by local resolution. Local resolution calculation was performed using blocres (1). View orientations as in Figure 1B.

(D) Angular distribution of particle projections. View orientations as in (C).
(E) FSC calculated between the model and the half map used for refinement (work), the other half map (free), and the full map.

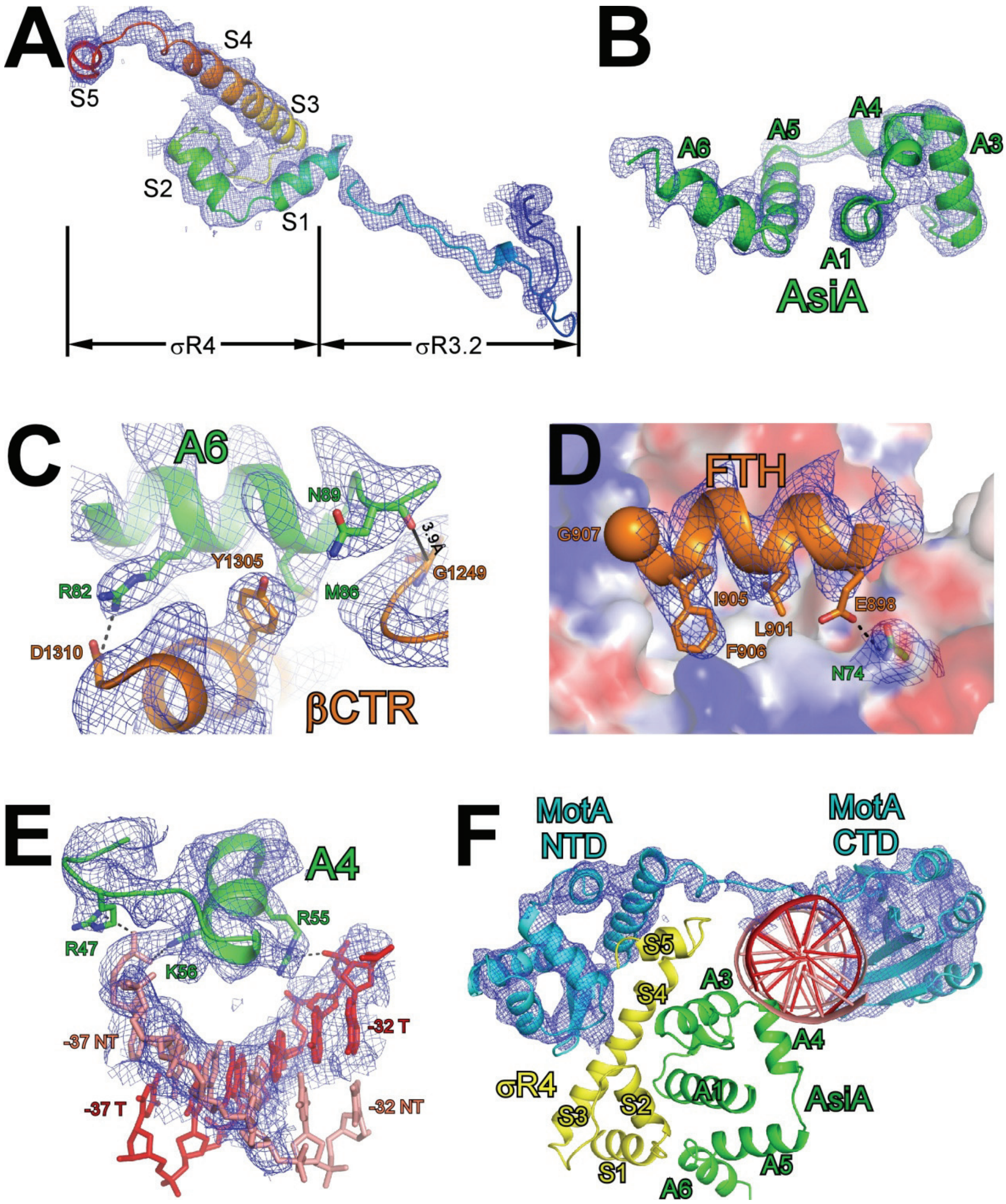


**Figure S4. Representative cryo-EM densities and superimposed models.**

(A) Cryo-EM density map (blue mesh) without B-factor sharpening and the superimposed model of σR3.2 and σR4. View orientation and colors as in the upper subpanel of Figure 2A.

(B) Cryo-EM density map (blue mesh) without B-factor sharpening and the superimposed model of AsiA. View orientation and colors as in Figure 3A.

(C) Cryo-EM density map (blue mesh) with B-factor sharpening and the superimposed model for the AsiA-βCTR interaction. View orientation and colors as in Figure 3C.

(D) Cryo-EM density map (blue mesh) with B-factor sharpening and the superimposed model for the AsiA-FTH interaction. View orientation and colors as in Figure 3D.

(E) Cryo-EM density map (blue mesh) with B-factor sharpening and the superimposed model for the AsiA-DNA interaction. View orientation and colors as in Figure 4A.

(F) Cryo-EM density map (blue mesh) without B-factor sharpening and the superimposed model of MotA. View orientation and colors as in the left subpanel of Figure 5A.

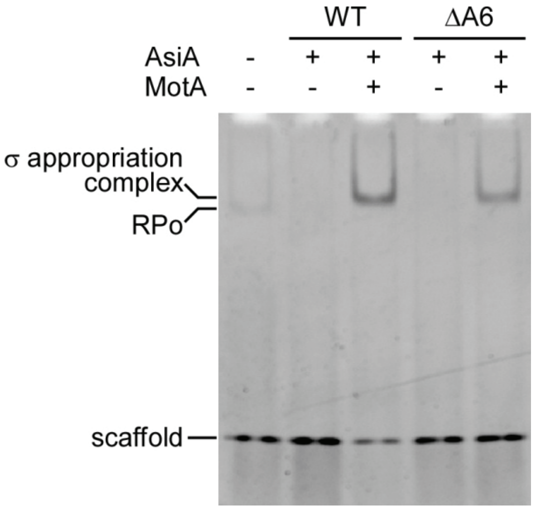


**Figure S5. Effects on formation of RPo and σ appropriation complex of deleting A6 (ΔA6).**

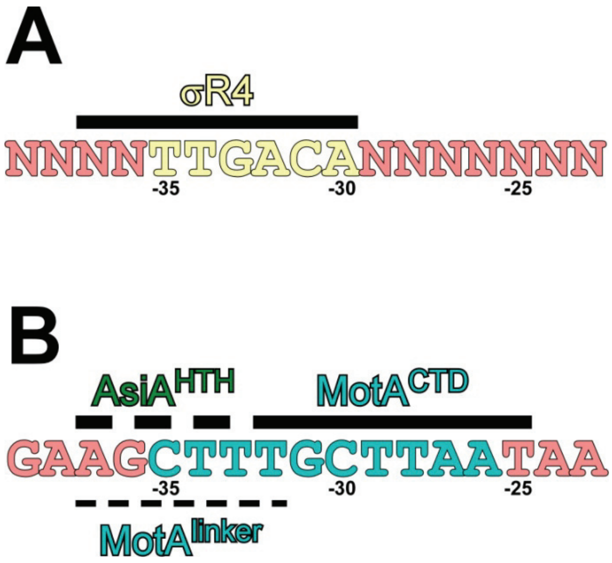


**Figure S6. The interaction of the upstream dsDNA in RPo (A) and σ appropriation complex (B), respectively.**

Nontemplate strand DNA sequence is shown. Yellow, σR4 and the consensus -35 element; cyan, MotA and the MotA box; green, AsiA. Thick solid line, sequence-specific interaction of the major groove; thick dashed line, non-sequence-specific interaction of the major groove; thin dashed line, non-sequence-specific interaction of the minor groove.

**Table S1. Cryo-EM data collection and refinement statistics.**

| | |
|---|---|
| **Data collection and processing** | |
| Microscope | Titan Krios |
| Voltage (kv) | 300 |
| Detector | K2 summit |
| Electron exposure (e/Å$^2$) | 56 |
| Defocus range (μm) | 1.5-2.5 |
| Data collection mode | Counting |
| Physical pixel size (Å/pixel) | 1.307 |
| Symmetry imposed | C1 |
| Initial particle images | 674,701 |
| Final particle images | 105,108 |
| Map resolution (Å)[a] | 3.79 |
| **Refinement** | |
| Map sharpening B-factor (Å$^2$) | -104 |
| Root-mean-square deviation | |
| Bond lengths (Å) | 0.008 |
| Bond angles (°) | 0.969 |
| Molprobity statistics | |
| Clashscore | 8.63 |
| Rotamer outliers (%) | 0.53 |
| Cβ outliers (%) | 0 |
| Ramachandran plot | |
| Favored (%) | 92.21 |
| Outliers (%) | 0.05 |

[a]Gold-standard FSC 0.143 cutoff criteria.

**Supplementary References**

1. Cardone, G., Heymann, J.B. and Steven, A.C. (2013) One number does not fit all: mapping local variations in resolution in cryo-EM reconstructions. *J. Struct. Biol.*, **184**, 226-236.
